# Interprofessional education in Erlangen: A needs analysis and the conceptual work of a student working group

**DOI:** 10.3205/zma001017

**Published:** 2016-04-29

**Authors:** Raffael Konietzko, Luca Frank, Nils Maudanz, Johannes Binder

**Affiliations:** 1 Friedrich-Alexander-Universität Erlangen-Nürnberg, Erlangen, Germany

**Keywords:** Interprofessionalism, current situation, students' perspective, project description, learning center, concept development

## Abstract

**Introduction:** Interprofessional education (IPE) is receiving growing significance both nationally and internationally. Despite this, organizational and curricular changes are posing challenges. The level of need for IPE and how changes can be made to curricula and infrastructure were investigated at the University of Erlangen in Germany.

**Method: **The student working group for interprofessional teaching (AGIL) has turned its attention to these issues. This group is composed of students from medicine, dentistry, molecular medicine, medical technology and speech therapy. In June, 2015, a needs analysis was carried out among the students in the study programs represented in the working group to assess the actual and target situation concerning IPE (n=1,105). In the search for answers and to better measure any needs, contact was sought with instructors.

**Results: **The majority of students feel that they are insufficiently educated in terms of interprofessional skills. A large proportion of the students wish to see expansion of the IPE offerings. Students also expressed a desire for additional spaces and welcomed the idea of an interprofessional learning center. AGIL began establishing interprofessional electives in October 2015. A concept for an interprofessional learning center was developed.

**Discussion:** Based on the survey results, a need for improvements to curricula and infrastructure can be seen; however, the results are limited to the student point of view. AGIL would like to establish more interprofessional electives. These courses would then facilitate curricular implementation. Modern ideas about study environments could be applied to IPE, in particular to promote informal forms of learning. Contact with instructors was crucial for the project work and should be expanded. Realizing and financing the learning center in Erlangen are now the future goals of AGIL. The aim is to create a foundation for this purpose.

## Zusammenfassung

Einleitung: Interprofessionelle Ausbildung (IPA) gewinnt national und international zunehmend an Bedeutung. Dennoch stellen organisatorische und curriculäre Umstrukturierung eine Herausforderung dar. Im Raum Erlangen soll untersucht werden, welchen Bedarf es an IPA gibt und wie curriculäre und infrastrukturelle Veränderungen angestrebt werden können.

**Methodik: **Dieser Fragen nimmt sich die studentische AG Interprofessionelle Lehre (AGIL) an. Diese setzt sich aus Studierenden der Human-, Zahn- und Molekularen Medizin, Medizintechnik und Logopädie zusammen. Im Juni 2015 wurde eine Bedarfsanalyse, zur Erhebung des Ist-Soll-Zustandes der IPA, unter den Studierenden der in der AG vertretenen Studiengängen durchgeführt (n=1105). Auf der Suche nach Lösungen und zur besseren Auslotung des Bedarfs wurde der Kontakt mit Dozenten gesucht.

**Ergebnisse:** Die Mehrheit der Studierenden fühlt sich in interprofessionellen Fähigkeiten unzureichend ausgebildet. Zu gleich wünscht sich der Großteil der Studierenden eine Ausweitung des Angebots an IPA. Die Studierenden sprechen sich zudem für zusätzliche Räumlichkeiten aus und befürworten ein interprofessionelles Lernzentrum. Die AGIL begann im Oktober 2015 mit der Etablierung interprofessioneller Wahlfächer. Es wurde ein Konzept eines interprofessionelles Lern- und Begegnungszentrum ausgearbeitet.

**Diskussion: **Aus den Ergebnissen der Umfrage kann ein Bedarf an curriculärer und infrastruktureller Verbesserung der IPA in Erlangen abgeleitet werden. Die Ergebnisse sind allerdings auf die studentische Sichtweise begrenzt. Die AGIL möchte weitere interprofessionelle Wahlfächer etablieren. Diese sollen die curriculäre Implementierung erleichtern. Moderne Konzepte zur Lernraumgestaltung könnten für IPA nutzbar gemacht werden, besonders um die informelle Form des Lernens zu fördern. Der Kontakt mit Dozenten stellt sich als essentiell für die Projektarbeit dar und soll ausgebaut werden. Die Verwirklichung und Finanzierung des Lernzentrums in Erlangen sind zukünftige Ziele der AGIL. Hierfür wird eine Stiftungsgründung angestrebt.

## Introduction

Our healthcare system is shaped by the interplay of different skilled professions ranging from academic professions to newly specialized programs for vocational study. All of these professionals perform their work with the goal of providing the best health care possible. To accomplish this, sharing and exchange between these groups is indispensible.

Internationally, interprofessional education (IPE) is becoming an increasingly permanent component of medical education. A good example of this can be found in the United States where approximately 90% of medical schools indicated in a survey that they offer interprofessional courses and clinical teaching. More than half had created an office or center specifically for IPE [1]. In Great Britain, IPE is found at 69% of the universities (n=113) that provide education in one or more health professions. Furthermore, 17% reported conducting research in this area [2]. According to the WHO, interprofessional collaboration is a promising solution for combating the global shortage of skilled labor in the health professions and primary care [3]. It has also been demonstrated that IPE positively influences the length of hospital stays and patient outcomes, and can even lead to lower costs for hospitals [4].

While initial experience is being gathered with IPE in Germany’s healthcare sector, the exchange between the health professions has not yet reached sufficient levels. The IPE committee of the Gesellschaft für Medizinische Ausbildung (GMA) is calling for the development of an overall philosophy for IPE. The problems which have been identified are primarily of an organizational or systemic nature. There is still much to learn, and those in research are only just starting to turn their attention to this field [5]. The German Council of Science and Humanities recommends designating IPE as one of five basic principles for restructuring medical education [6]. The National Competency-based Catalogue of Learning Objectives for Undergraduate Medical Education dedicates an entire chapter to IPE [http://www.nklm.de].

The necessity of fulfilling organizational, infrastructural and curricular pre-requisites to successfully implement IPE is constantly being pointed out, and was already taken into consideration by the OECD in its concept of a “health university” in 1977 [7]. Health universities are to offer integrated interprofessional education oriented toward local healthcare needs. This concept represents an ideal situation that, according to a review on the development of the health universities, appears difficult to achieve [8]. In terms of health universities, infrastructure has been created to varying extents in Germany. Consequently, there are interprofessional skills labs, such as the interdisciplinary medical training center in Dresden [http://tu-dresden.de/die_tu_dresden/fakultaeten/medizinische_fakultaet/inst/mpz], as well as entire campuses dedicated to interprofessional learning such as the Hochschule für Gesundheit in Bochum [http://www.hs-gesundheit.de/de/thema/die-hochschule/]. While on a campus of this nature most of the education takes place at one location, in skills labs student learn practical and communication skills only in connection with specific segments or parts of the particular educational program.

To date, interprofessional collaboration has taken place at the medical school of the Friedrich Alexander University in Erlangen-Nuremberg (FAU) only in individual instances. Joint courses in different fields are offered (e.g. dissection course with dental and medical students, imaging courses with medical students and students studying medical process management). Aside from these isolated points of intersection, this kind of interdisciplinary communication appears otherwise absent from the curricula. There is still great potential within the infrastructure for accommodating IPE in Erlangen. Based on these considerations the following issues come into focus for Erlangen:

1. Current situation: Is the need for IPE met in Erlangen from the student perspective?
2. Target situation: Do students desire more IPE in their education?
3. Which concepts pertaining to curriculum and infrastructure could be used to implement IPE in Erlangen?

To address these questions, the student working group on interprofessional teaching (AGIL) was created in December 2014. AGIL presently consists of students pursuing studies in medicine, dentistry, molecular medicine, medical technology, and speech therapy. These students have all completed varying numbers of semesters.

## Method

During weekly meetings, an atmosphere for effective project work was created with the help of soft skills training. In May, 2015, a planning meeting was held to generate a definitive timeline for accomplishing tasks. The questions above were first answered in independent subgroups of the working group. A leader was appointed for each subgroup.

To respond to questions 1 and 2, the subgroup dealing with evaluation surveyed students in medicine, medical technology, dentistry, and molecular medicine in June 2015. In addition, data was gathered regarding the current student take on IPE. The student surveys asked about the exchange between the health professions and the current study space on offer. Students in the clinical phase of their education also gave their assessment regarding the existence of selected interprofessional core competencies [9]. Opinions on new approaches in medical education were asked for. The student surveys were drafted using the software “Umfrage Online”, and the items reflected six-point Likert scales. Students were invited to participate in the survey via FaceBook and email lists administered by the representative student bodies. Following a period of three weeks student access to the survey was cut off.

To find answers to the third question, three other subgroups grappled with possible solutions. Functioning as an information point of contact within the larger working group, the subgroup dealing with *research* continued to compile information on the academic context of IPE. The subgroup concerned with *academic courses* developed (extra-)curricular electives and required electives, while the subgroup addressing concept worked on infrastructural approaches to meeting an increased need for IPE.

In addition, the working group took on the responsibility of establishing contact with university instructors to raise awareness about IPE and to work on concrete improvements for Erlangen. This was crucial to ensure the needed support of the faculty to sustainably realize such a project as this one. For the purpose of defining the later level of engagement, three different activity profiles were set down: consulting, establishing academic course offerings, and participating on a task force. The task force assumes responsibility for elaborating on the legal and economic aspects of the curricular and infrastructural concepts.

## Results

The survey results were analyzed in terms of the defined issues and the following presents the results for each study program accompanied by a summary.

**Figure 1:**
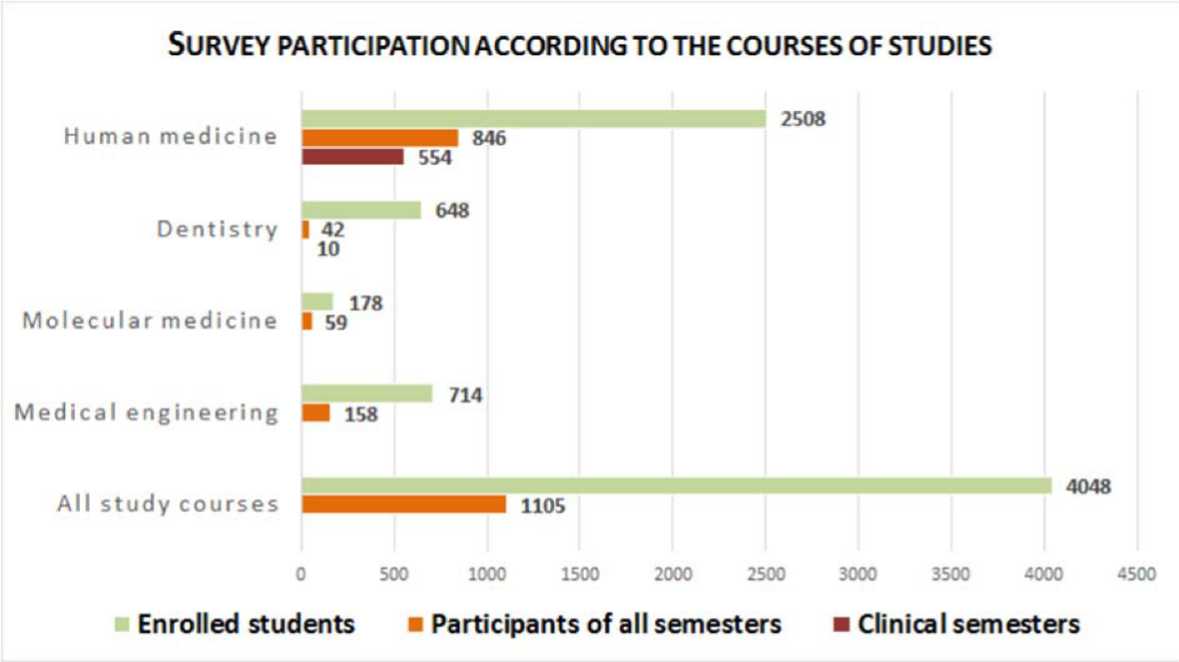
Survey participation according to courses of studies. Participants of human medicine and dentistry are divided into clinical years and students from all years. The highest participation rate was reached in human medicine. Followed by molecular medicine, medical technology and dentistry with lowest participation. Over all participation rate was 27%.

A total of 846 medical students, 158 medical technology students, 59 molecular medicine students, and 42 dental students responded to the survey, meaning that 1,105 of 4,048 students (27.3%) took part (see figure 1 (Fig. 1)).

**Figure 2:**
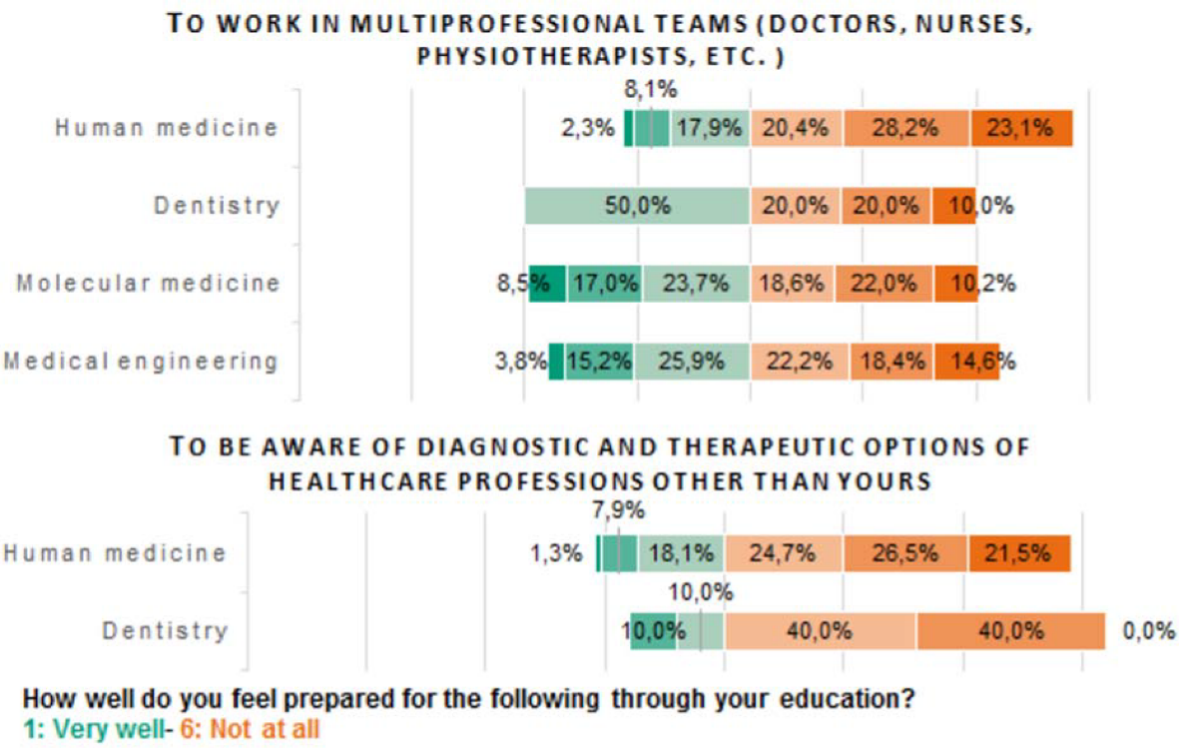
Data to evaluate the current situation of IPE in Erlangen from a students‘ perspective. For human medicine and dentistry only students in clinical years participated. Question 2 was not answered by students of medical technology and molecular medicine. The majority of the students of medical technology, human medicine and molecular medicine does not feel well prepared for the work in multiprofessional teams through their education. Furthermore, the majority of students of human medicine does not feel aware of diagnostic and therapeutic options of professions other than theirs.

Issue 1:

To investigate the need for IPE in Erlangen, students in the clinical phase of study (medicine: n=554; dentistry: n=10) and at all semester levels (molecular medicine: n=59; medical technology: n=158) were surveyed in regard to interprofessional core competencies (see figure 2 (Fig. 2)). A total of 781 out of 3,033 students (25.8%) participated in this component:

- The majority of the students surveyed (excepting dental students with 50%) felt their education was rather not preparing them well for work in multiprofessional teams (medicine: 71.7%; molecular medicine: 50.8%: medical technology: 55.2%).
- During their later careers, medical and dental practitioners are most directly involved in patient care. Of these students, most feel themselves rather unprepared to take the diagnoses and therapy options used in other health professions into account (medicine: 72.7%; dentistry: 80%).

**Figure 3:**
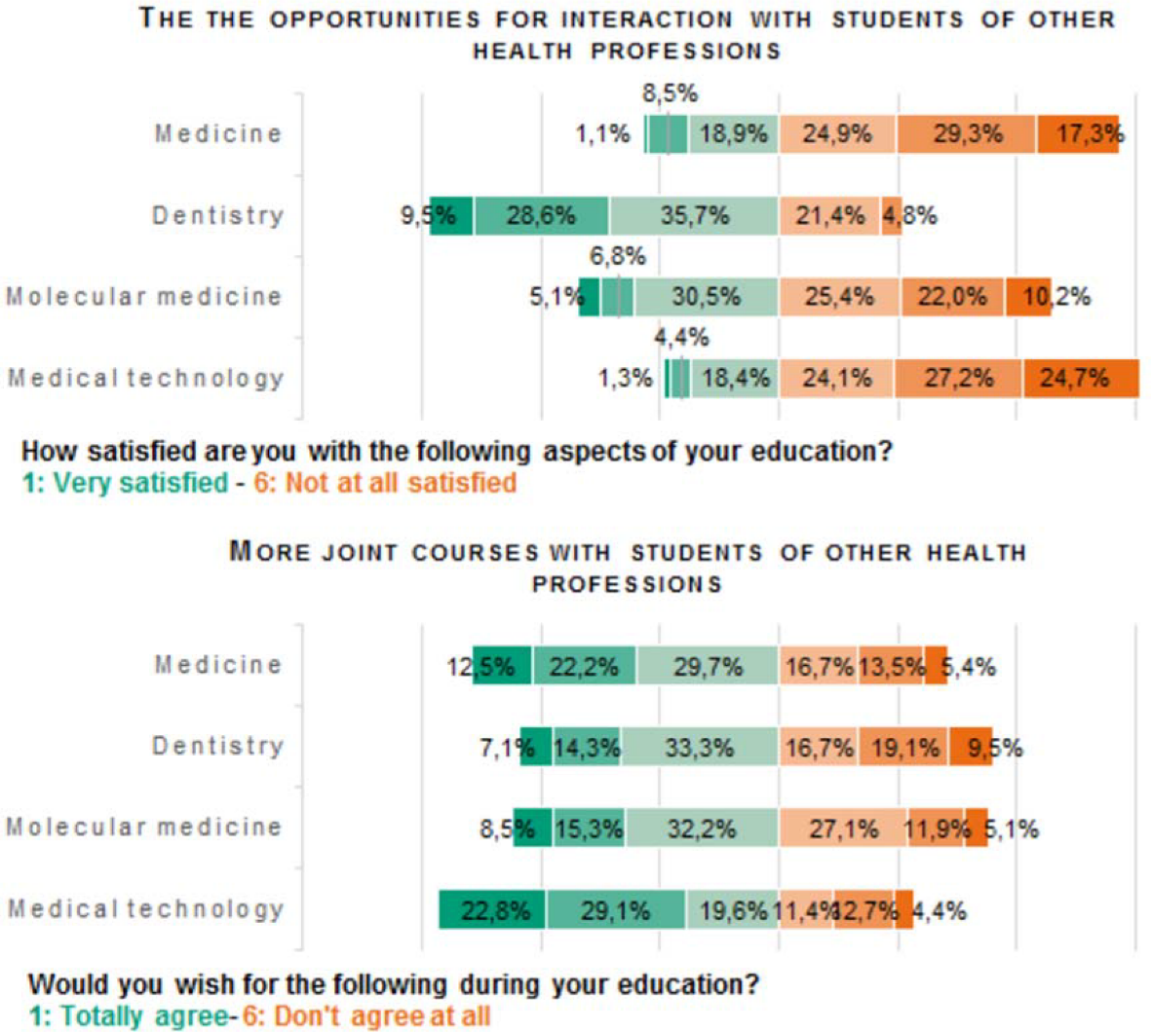
Data of the target situation of IPE in Erlangen from a students‘ perspective. All students of all years participated in this section of the evaluation (n=1105). The majority of the students of medical technology, human medicine and molecular medicine is rather unsatisfied with the possibilities of exchange with students of other health professions during their education. The majority of all four courses of studies wishes for more joint courses with other health professions.

Issue 2:

To investigate if students desire more IPE, all survey participants (n=1,105) were asked about their preferences (see figure 3 (Fig. 3)):

- The majority of those surveyed (with the exception of dental students with 26.2%) are rather dissatisfied with the opportunities for interaction with students of other health professions (medicine: 71.5%; molecular medicine: 57.6%; medical technology: 76.0%)
- A large percentage of students wish for more joint courses with students of other health professions (medicine: 64.4%; dentistry: 54.7%; molecular medicine: 56.0%; medical technology: 71.5%).

Issue 3:

Based on the information gathered, the subgroup within the working group dedicated to academic courses intends to expand the IPE course offerings, specifically in terms of electives. In October, 2015, case history groups were introduced for students of psychology, speech therapy, dentistry and medicine. The intention has been to offer a seminar in implantology for dental, medical, and medical technology students starting in August 2016. Contact has already been established with sponsors to finance this seminar.

As a basic pillar for implementing IPE in Erlangen, an interprofessional learning center for medical education (ILMA) has been envisioned to serve as a central meeting point on campus for students of medicine and the other health professions. Specifically, skills labs and spaces for study and recreation are to be included in an attempt to push the ILMA beyond the basic concept of the interprofessional skills lab. Characteristics of an integrated campus are partially embraced: the housing of (extra-)curricular courses at the learning center will be limited to IPE. Above all, this learning center is to be a place for individual study. Exchange between students can, however, be a self-evident component of studying and free-time activities. With the current research undertaken at the university and later career options presented at one central point of contact, an atmosphere for innovative joint projects can also be created.

**Figure 4:**
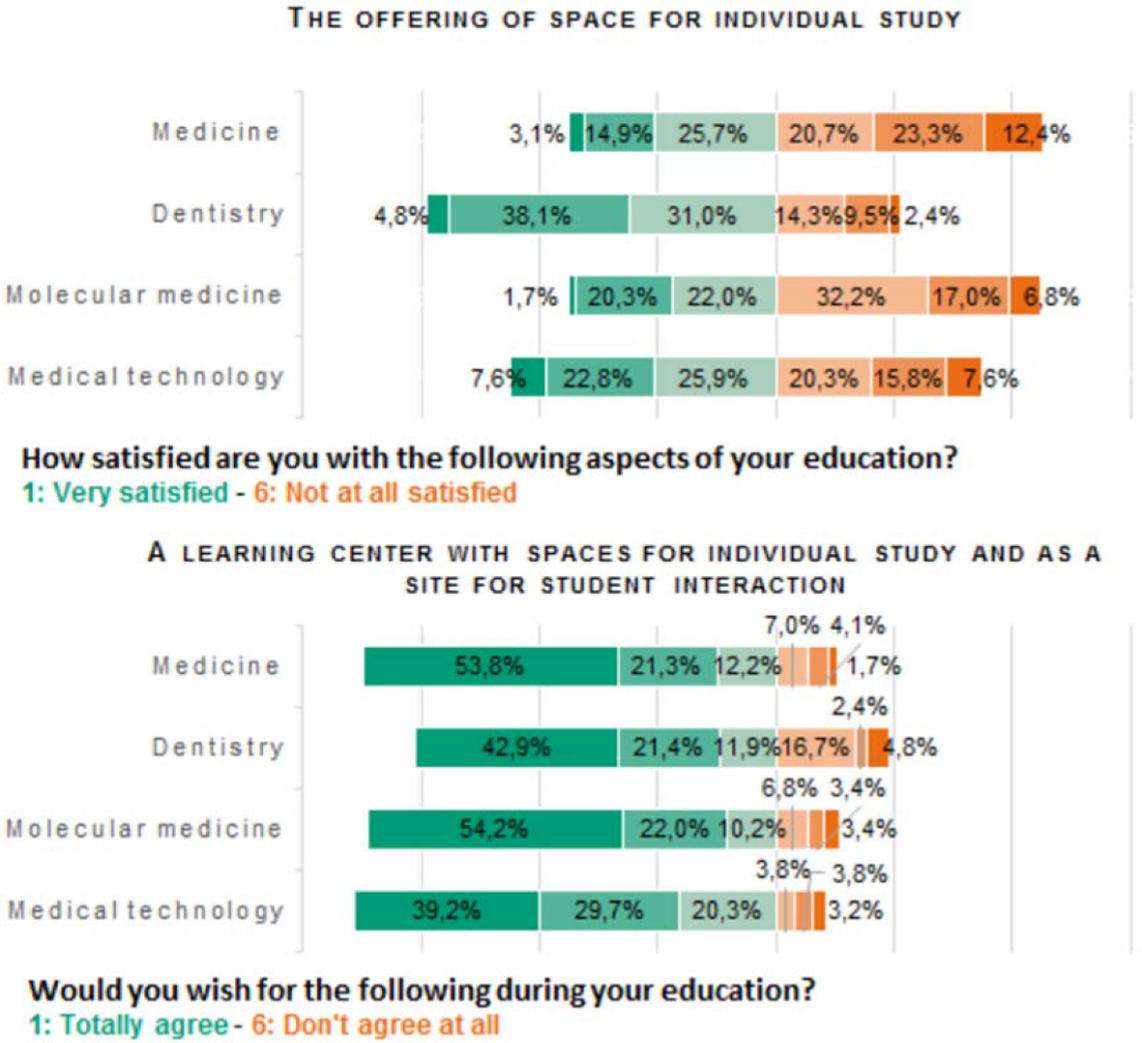
Demand and acceptance of a concrete solution. Students of all years participated in this section of the evaluation (n=1105). The majority of students of human medicine and molecular medicine are rather dissatisfied with the offering of space for individual study. The majority of all students wishes for a central learning center with place for individual study and as a place for student interaction.

To determine student need for such a learning center, all survey participants were asked about the currently available spaces in Erlangen (see figure 4 (Fig. 4)):

- The spaces at the university presently available for individual study are assessed differently. Students of medicine (56.4%) and molecular medicine (56.0%) are most likely to assess the situation as poor. Even a substantial percentage of medical technology students are dissatisfied with the situation (43.7%). Only dental students are satisfied for the most part (73.9%).
- The majority of students desire a learning center with spaces for individual study and as a site for student interaction (medicine: 87.3%; dentistry: 76.2%; molecular medicine: 86.4%, medical technology: 89.2%).

Most instructors indicated a willingness to cooperate: 15 of the 25 instructors initially contacted responded to AGIL. Thirteen of these are able to envision being involved in general cooperation, with six of these serving as active members of the task force.

## Discussion

The needs analysis presented here reveals several strengths and weaknesses. The time point of the survey can be regarded positively. AGIL had set itself the goal of raising awareness of IPE in Erlangen through public relations. To avoid falsification of the survey results, the survey was conducted prior to beginning any publicity measures. The survey’s response rate of 27% appears to be representative at first glance; however, the response rates for the separate study programs must be critically assessed. The low participation of dental students, particularly of those in the clinical phase of study (n=10), could lead to a bias in the results. Giving too much weight to this detail in the interpretation of the results should therefore be avoided. Furthermore, it must be noted that the method applied only accounts for student opinions. Only the dialogues held with instructors were able to roughly mirror the student point of view, something that is to be objectified in another survey. Since the three questions are specifically formulated to elicit the student point of view, the results of the survey was able supply answers to these.

Issue 1:

Although practicing physicians, as the central caregivers, always work in tandem with other health professions, they are very likely to regard themselves as poorly prepared by their education to deal with the additional diagnoses and therapy options offered by these other health professions (72.7%). Unfortunately, the results seen here for dental students cannot be viewed as representative (see above). Despite this, a need for more IPE can be inferred since the majority of respondents feel ill prepared for interprofessional collaboration in their later careers (66.5%). Due to the fact that at the University of Erlangen hardly any content regarding clinical practice is taught during the preclinical phase of study, an analysis of the current situation regarding the core competencies of medical and dental students at this level of study was not undertaken.

Issue 2:

Although, based on the results, IPE appears to be relatively well established in dentistry, the low rate of response also limits the ability to interpret the results. Furthermore, the majority of the other three study programs voiced dissatisfaction with the opportunities for interaction with students of other health professions (71.4%). At the same time, the majority from all study programs desired more joint courses with students of other health professions (64.6%).

Issue 3:

The analysis of the actual and target situations resulting from questions 1 and 2 shows that IPE should be more broadly present in study programs. However, it is impossible to conclude from the results how this should happen. The creation of new (required) elective subjects by the subgroup for *academic courses* is necessary. This will allow for piloting to provide experience to identify the best curricular options for implementing IPE. Ultimately, this must take place to make IPE accessible to all students. A further responsibility of the subgroup evaluation will be monitoring these (required) elective courses.

The situation regarding space was evaluated negatively by a large part of the students. Only dental students seem to be satisfied for the most part. This could be traced to the fact that a large part of the teaching is done in a well equipped teaching hospital. A central medical learning center, in alignment with the vision of ILMA, was positively seen by all students regardless of major focus.

A comprehensive concept for a health campus is certainly offered by the model of the health university. Partial aspects of the health university could be applied to our concept. In the review by Sottas et al mentioned in the introduction, spatial design, architecture and the logistics of patient management are given a major role [8]. According to the German initiative for network information, there are variables critical to the process of developing learning spaces and to education that make it at all possible for students to develop competencies. The learning and study spaces of the future should provide inspiration, take social and communicative needs into account, and promote the practical implementation and application of what is learned [10]. This recommendation may be directed toward libraries, but insights and knowledge for planning the learning center could also be gleaned from it. Moreover, a systematic review of Best Evidence in Medical Education (BEME) - Collaboration describes that the interaction outside of class time, meaning in short breaks during seminars and lectures and during free-shared free-time activities, can lead to positive attitudes toward each other among those in the differing health professions and cement formal learning [11]. This is also referred to as informal learning and occurs mostly outside of scheduled courses. Precisely the combination of spaces with room for learning and skills labs could be of interest for IPE in terms of this form of learning.

The center must be sufficiently equipped functionally and integrated into the university. Very significant in this regard were the dialogues with the instructors. For, only by doing this it was possible to bring attention to IPE. The criticism that was expressed in these conversations was essential for honing the overall concept. At the same time, the support shown by the instructors confirmed our previous decisions and actions. In the future, AGIL will continue to draw on university instructors as cooperative partners and important sources of information. A newly founded society to promote IPE could function as a contact partner for both university and off-campus interests, provide incentive to establish IPE, and facilitate the financing of courses. The aim is ultimately the creation of a foundation to finance the learning center.

Whether or not such a center would induce students to engage in individual learning and improve contact between different health professions must be investigated. The extent to which the conception of such a center can support education research is also of interest. The question is also raised as to whether or not the basis for interprofessional research projects is created, meaning that there are qualitative effects for the university as an academic institution. Not least, the center could serve as a central point of convergence for university and off-campus institutions.

## Conclusion

Conducting the evaluation illustrated the need for IPE and infrastructure and laid the foundation for future evaluations of the project’s influence. The working group was able to come up with ideas to improve the situation in Erlangen that also met with instructor approval. International insights on IPE and modern recommendations for designing study environments suggest an approach that simultaneously changes curriculum and provides state-of-the-art learning space. In light of the evaluation results, the information gathered from speaking with university instructors and the national and international recommendations, AGIL has taken on the curricular implementation of IPE and the realization and financing of the new learning center as its new project goals.

Over the long term IPE should become established across Germany. Building a network for the purpose of sharing information could be useful. AGIL is ready to share its knowledge and experiences with other student working groups.

## Competing interests

The authors declare that they have no competing interests.
